# Effects of Sudden Drop in Salinity on Osmotic Pressure Regulation and Antioxidant Defense Mechanism of *Scapharca subcrenata*

**DOI:** 10.3389/fphys.2020.00884

**Published:** 2020-07-17

**Authors:** Zhang Mo, Li Li, Liu Ying, Gao Xiaolong

**Affiliations:** ^1^State Key Laboratory of Marine Environmental Science, College of Ocean and Earth Sciences, Xiamen University, Xiamen, China; ^2^Key Laboratory of Experimental Marine Biology, Institute of Oceanology, Chinese Academy of Sciences, Qingdao, China; ^3^Marine Biology Institute of Shandong Province, Qingdao, China; ^4^College of Marine Technology and Environment, Dalian Ocean University, Dalian, China

**Keywords:** *Scapharca subcrenata*, salinity, osmotic pressure, reactive oxygen species, antioxidant defense, heat shock proteins, cathepsin

## Abstract

Salinity is an important ecological factor that impacts the growth and survival of aquatic organisms. The salinity of seawater in coastal and estuarine areas is often subject to dynamic changes because of seasonal rainfall and continental runoff. Thus, the current study investigated the effects of sudden changes in salinity on the survival rate and osmotic pressure regulation mechanisms of bottom-sowing seedlings of the economically important ark shell, *Scapharca subcrenata*. By simulating the sudden changes that occur in seawater salinity after rainstorms, the results showed that the osmotic pressure of the hemolymph and Na^+^, K^+^, Ca^2+^, and Cl^–^ concentrations first decreased and then increased. When the salinity decreased from 30 to 14‰, hemoglobin, soluble total protein, taurine, and total free amino acid gradually increased; maximum levels of hemoglobin, soluble total protein, and taurine occurred once the salinity increased to 22‰ at 96 h. After 96 h, the total free amino acid content increased until 144 h. The reactive oxygen species (ROS) content and total antioxidant capacity (T-AOC) peaked at 96 h, whereas the expression levels of Mn-superoxide dismutase (*MnSOD*) and catalase (*CAT*) increased earlier, indicating that, with continuous ROS generation, antioxidant defense mechanisms were activated to avoid oxidative damage. Expression levels of cathepsin C (*CTSC*), cathepsin D (*CTSD*), heat shock protein 20 (*HSP20*), and heat shock protein 70 (*HSP70*) were significantly higher than in the control group at 48 h (salinity level 14‰); the expression levels of *HSP20*, heat shock protein 90 (*HSP90*), *MnSOD*, and glutathione peroxidase (*GP*_*x*_) remained high, indicating that they were still required for osmotic pressure regulation to maintain the dynamic balance between the generation and removal of ROS as the salinity level increased. These results not only add to our basic understanding of the aquatic ecology of *S. subcrenata*, but also provide a theoretical ground for improving the survival rate of bottom-sowing, propagation, and release of *S. subcrenata* seedlings.

## Introduction

In coastal areas, salinity is subject to drastic changes in response to seasonal precipitation, tides, ocean currents, and seawater evaporation (Huong et al., 2010; Johnson et al., 2011). In response to changes in environmental salinity, organisms might perceive changes in osmotic pressure, responding metabolically to regulate the ion concentration and composition in cells, to maintain an optimal metabolic state. The formation of this optimal state is reflected in the dynamic equilibrium of internal osmotic pressure through physiological responses and the secretion and absorption of salt and water by the body (Kültz, 2012).

The ark shell *Scapharca subcrenata* is an economically important marine shellfish in China; it is vertically distributed in the sea between the low tide line and a 7-m depth. Most individuals live in fresh water-affected inner bay and neritic regions, showing a range of adaptations to salinity, and preferring to inhabit soft mud or muddy seafloors with a high sand content (Wang and Wang, 2008). Recently, overfishing and environmental changes have led to a decrease in available habitats. To restore the declining habitats of *S. subcrenata*, large-scale enhancements and release of seeds have occurred. In estuarine or intertidal zones, which are subject to significant changes in salinity, organisms with a fixed living mode or those only able to move slowly can grow well within an appropriate range of salinity; however, if the habitat salinity increases or decreases beyond that range, their growth is impacted and can even lead to a stress response, which in turn diminishes immune resistance (Wang and Chen, 2005; Sung et al., 2011). Therefore, selecting a suitable release area based on the salinity tolerance mechanisms of *S. subcrenata* would help improve the survival rate of *S. subcrenata* seeds.

Euryhaline organisms are better able to regulate their osmotic pressure compared with stenohaline organisms, enabling them to maintain a higher rate of food intake, absorption, transformation, and growth efficiency in a wider salinity range (Tseng and Hwang, 2008). However, the optimal growth salinity varies significantly from species to species. When the osmotic pressure of water is similar to the internal osmotic pressure, the energy consumption of the organism allocated to the regulation of internal osmotic pressure is the lowest, but is most beneficial to survival and growth (Verbalis, 2007; Seale et al., 2012). After *Litopenaeus vannamei* fed under a salinity of 35 psu was transferred to, and kept in 25, 20, or 15 psu environments for 1–6 h, the activity of granule cells, blood cells, phenol oxidase, and superoxide dismutase (SOD) significantly decreased. Subject to the dual-factor stress of *Vibrio alginolyticus* and salinity dip, the above immune indexes decreased more significantly, suggesting that the immunity level of *L. vannamei* also significantly decreased (Lin et al., 2012). When *Takifugu obscurus* was placed under a salinity of 30 psu, the number of chloride-secreting cells in the gill filament increased, as did its volume; in addition, hormones, such as growth hormone and cortisol, triggered the synthesis of more transport proteins to stimulate the proliferation and differentiation of chlorine cells, thereby changing the transmembrane transport capacity of ions and water molecules (Wang et al., 2016). Zhang et al. (2018) performed transcriptome sequencing on *Apostichopus japonicas* grown under 20 and 30 psu and identified 109 differentially expressed genes. Gao et al. (2017a) simulated the falling then rising trend of seawater salinity after a rainstorm and found that the expression levels of *CAT*, *TP*_*x*_, *GST*_*s*_, *GST*_*m*_ tended to rise then fall, and these were significantly higher than groups with constant salinity.

Consequently, osmotic pressure regulation mechanisms not only involve the transport of ions and water and the regulation of the neuroendocrine system, but also depend on energy metabolism to maintain the activity of transport proteins and on the auxiliary functions of different kinds of osmotic pressure-regulating molecules, such as free amino acids. To date, few reports have focused on the salinity tolerance range of *S. subcrenata* and the osmotic pressure regulation mechanisms involved, despite the economic importance of this species. Therefore, the results of this study that shed light on the physiological adaptation mechanisms of *S. subcrenata* to changes in salinity will help increase not only our basic understanding of the biology of *S. subcrenata*, but also the survival rate of bottom-sown seedlings in habitats subject to regular changes in salinity.

## Materials and Methods

### Source and Acclimation of *S. subcrenata*

*Scapharca subcrenata* (shell length: 32.85 ± 1.73 mm, body weight: 8.15 ± 1.03 g) were purchased from Fuyuan fisheries company (Rizhao, Shandong, China), and all experimental *S. subcrenata* were sourced from the same batch after artificial hatching. After purchasing the *S. subcrenatas*, they were acclimated in one culture container (length 1.2 m × width 1 m × height 1 m, water volume: 1200 L) for 15 days; water temperature was kept at 22°C, salinity at 30 + 1, pH at 7.9, dissolved oxygen concentration at > 6 mg/L, and the light cycle was set as the natural light cycle. Aquaculture water was obtained from the natural sea area and used after sedimentation and sand filtration. Two-thirds of the water was replaced with fresh seawater each day at 09:00 to ensure good water quality. During the period of acclimation, the food mixture of *Chlorella vulgaris*, *Isochrysis galbana*, and *Platymonas subcordiformis* was fed once a day at a volume ratio of 1:1:1 and then the food concentration was measured every 6 h.

### Experimental Design

**FIGURE 1:**
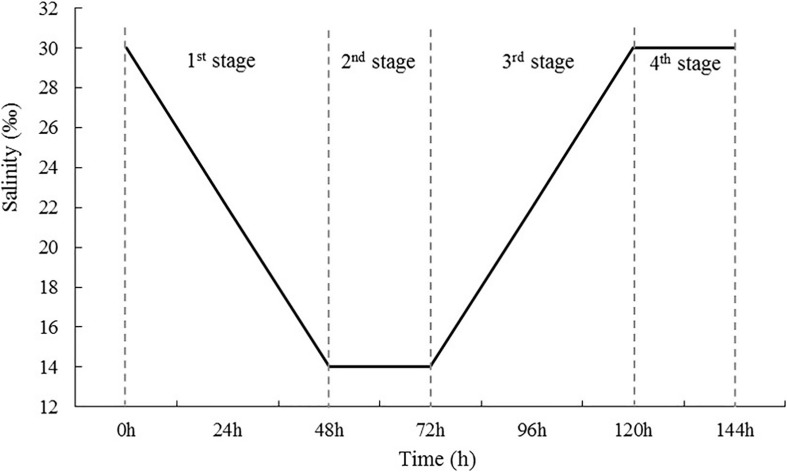
Diagram of the salinity change mode used in the experiment. First, salinity decreased gradually from 30 to 14 psu at a rate of 8 psu every 24 h (first stage), and then was maintained at low levels for 24 h (second stage). After 72-h stress, salinity increased gradually to 30 psu at a rate of 8 psu 24 h^–1^ (third stage) and was then maintained at 30 psu for 24 h (fourth stage).

In total, 200 *S. subcrenatas* were randomly selected from the culture container and divided among five aquarium tanks (0.6 m × 0.5 m × 0.6 m, tank volume: 180 L), with 40 per tank. Four of the tanks were used to simulate four phases in the changes in seawater salinity after rainfall (Figure 1); four replicates were used. In Phase 1, the salinity was reduced from 30 to 14‰ at a rate of eight levels every 24 h; in Phase 2, the salinity was kept at 14‰ for 24 h; in Phase 3, the salinity was increased from 14 to 30‰ at a rate of eight levels every 24 h; and in Phase 4, the salinity was maintained at 30‰ for 24 h. Meanwhile, *S. subcrenata* cultured at a salinity of 30‰ in an aquarium was used as the control group, in which the salinity stayed at the same level throughout the study. The low-salinity seawater was prepared using tap water with 24 h aeration and seawater prepared through natural sand filtration; while the high-salinity seawater was prepared by adding sea salts (Blue Treasure Co., Ltd., Qingdao, China) to the experiment group in low-salinity seawater.

All *S. subcrenatas* were starved for 24 h prior to the experiment. During the experiment, no feed was given and the room temperature was kept at 22°C with air-conditioning control, with the same pH as it was in acclimation. Each aquarium was provided with continuous aeration to maintain the concentration of dissolved oxygen above 6 mg/L, and the ammonia concentration was always below 0.1 mg/L throughout the experiment.

### Collection of Samples

Samples of gill tissue and hemolymph were collected at different time points in each phase: Phase 1, at 24 and 48 h after the start of the experiment, when salinity was reduced to 22‰ (DS1) and 14‰ (DS2); Phase 2, at 72 h after the beginning of experiment, when the salinity was maintained at 14‰ for 24 h (DL1); Phase 3, at 96 and 120 h after the start of experiment, when the salinity was increased to 22‰ (IS1) and 30‰ (IS2), respectively; and Phase 4, at 144 h, when the salinity was maintained at 30‰ for 24 h (IL1).

Before the experiment, 10 *S. subcrenatas* were randomly selected as the samples at 0 h for later analysis. The survival rate of *S. subcrenatas* in each aquarium was recorded at 24, 48, 72, 96, 120, and 144 h after the beginning of the experiment, and three *S. subcrenatas* were randomly selected from each aquarium, giving 15 *S. subcrenatas* in total, to measure the hemolymph osmotic pressure. The concentration of Na^+^, K^+^, Ca^2+^, Cl^–^, hemoglobin, soluble protein, and the concentration of taurine and total free amino acids were determined after centrifugation of hemolymph. Gill tissues were taken to analyze and determine the enzyme activity of Na^+^/K^+^-ATPase, the content of reactive oxygen species (ROS), total antioxidant capacity (T-AOC), and the gene expression of Mn-SOD (*MnSOD*), Catalase (*CAT*), glutathione peroxidase (*GP*_*x*_), cathepsin C (*CTSC*), cathepsin D (*CTSD*), heat shock protein 20 (*HSP20*), *HSP70*, and *HSP90*.

### Assay of Samples

#### Assay of Physiological Indices in Hemolymph

At the end of Phase 4, each *S. subcrenatas* was euthanized and dissected; the adductor muscle was removed and hemolymph was extracted from the blood sinus using a 10-mL medical syringe. It was then placed it in a 1.5-mL sterile centrifuge tube in an ice bath. The time between the removal of any sample and the completion of sampling process was always < 10 min. Each centrifuge tube contained the hemolymph of five individuals. A Fiske 210 Micro-Sample Osmometer (Advanced Instruments, Norwood, MA, United States) was used for the determination of osmotic pressure. The remainder of the hemolymph was centrifuged at 4°C, 10,000 × *g*/min for 10 min, and the supernatant was used to determine the concentration of hemocyanin, total soluble protein, Na^+^, K^+^, Ca^2+^, Cl^–^, taurine, and total free amino acids.

The concentration of hemoglobin was measured using the hemiglobincyanide (HiCN) assay, and the contents of hemoglobin were measured with a hemoglobin instrument (WJX-1; Shanghai Touching Technology Co., Shanghai, China).

The concentration of total soluble protein in these samples was calculated using Bradford’s (1976) Coomassie Brilliant Blue, with bovine serum albumin as the standard protein.

The contents of Na^+^, K^+^, Ca^2+^, and Cl^–^ in the supernatant of hemolymph under each salinity level were determined with a kit from the Nanjing Jiancheng Bioengineering Research Institute (Nanjing, China) (Gao et al., 2017a).

The mixed hemolymph supernatant was analyzed at Qingdao Institute of Bioenergy and Bioprocess Technology, Chinese Academy of Sciences (Qingdao, China). After being treated with 8% trichloroacetic acid and sonication for 20 min, the supernatant was collected and tested under Sykam S-433D Automated Amino Acid Analyzer (Munich, Germany) to determine the concentration of taurine and total free amino acids in the hemolymph samples, respectively.

#### Na^+^/K^+^-ATPase Activity, ROS Content, and T-AOC Assays

After the hemolymph had been collected, the gill tissues were cut with scissors on an ice tray and evenly divided into two sections; each section was placed into a 1.5 mL centrifuge tube, quickly placed into liquid nitrogen, and then transferred to a –80°C refrigerator until use. Then 0.2–0.4 g gill tissues were extracted, and fully ground with the addition of 1.8 mL 0.86% saline in the ice-water bath. After grinding, the material was centrifuged at 3500 × *g*/min for 10 min until 10% tissue homogenates were obtained, to determine the content of ROS and T-AOC. The activity of Na^+^/K^+^-ATPase was determined using a kit supplied by Nanjing Jiancheng Bioengineering Institute and the enzyme activity was determined using the phosphorus determination method (Kaplan, 2005). One unit of enzyme activity was defined as the generation of 1 μmol inorganic phosphate from the decomposition of ATP per minute by ATPase in 1 mg of tissue protein (Peterson, 1978). The protein content in muscle homogenates was determined using Bradford’s (1976) Coomassie Brilliant Blue, with bovine serum albumin as the standard protein.

Regarding the ROS content, three gill tissue homogenates from each aquarium were centrifuged at 1000 × *g* for 10 min, and then 1 mmol/L DCFH-DA (2′,7′-dichlorofluorescin diacetate) was added to the supernatant. The mixture was incubated at 37°C for 30 min after being fully and evenly mixed. A fluorescence spectrophotometer (960MC, INESA. CC) was used to determine the fluorescence intensity at the optimum excitation wavelength of 500 nm and at the optimum emission wavelength of 525 nm, and the results were expressed as fluorescence arbitrary units (A.U.) (Gao et al., 2017b).

Total antioxidant capacity was determined using the ferric reducing ability of plasma method (Benzie and Strain, 1996). At 37°C, a unit (U/mg) of T-AOC was defined as the increase of 0.01 of optical density of all chemical reaction substances by 1 mg of tissue protein per minute.

Finally, the rest of the muscle tissues were absorbed with a filter paper, and the wet weight of muscle tissues for fifteen individuals was measured at each time point. These tissues were dried to constant weight in a 202-00S oven (Lichen Co. Ltd., Shanghai, China) at 105°C, their dry weight was measured, and the moisture content calculated. Muscle moisture = (wet weight of muscle tissues – dry weight of muscle tissues)/wet weight of muscle tissues × 100%.

#### Analysis of Gene Expression

A sample of gill tissue was added to a mortar and ground with liquid nitrogen. Then, 0.05 mg of the powder obtained was quickly mixed with 1 mL TRIzol (Invitrogen, United States) to extract the total RNA from the gill tissue. Total RNA was extracted by removing the residual DNA from the sample using RQI RNase-Free DNase (TaKaRa, Kusatsu, Japan), and then RNA was reverse transcribed to cDNA using M-MLV reverse transcriptase (Promega, Madison, WI, United States). Real-time quantitative PCR was conducted using the SYBR^®^ Premix Ex Taq^TM^ II kit (Tli RNaseH Plus) (TaKaRa) and the TaKaRa Thermal Cycler Dice^TM^ Real Time System TP800 instrument. The specific primers were designed based on cDNA complete sequences submitted to GenBank, and *MnSOD*, *CAT*, *GP*_*x*_, *CTSC*, *CTSD*, *HSP20*, *HSP70*, *HSP90*, and the reference gene β-actin were analyzed. Genetic information and primer sequences are presented in Table 1.

**TABLE 1:** Real-time quantitative PCR primers for antioxidant enzymes, cathepsin, and heat shock proteins’ genes of *Scapharca subcrenata*.

**Gene**	**Sequence (5′–3′)**	**Size (bp)**	**Efficiency (%)**	**References**
Mn-SOD	F: CGGAACCACTCCTCCGTCA	167	100.15	Zheng et al., 2015
	R: ACGCCGATTTTCTAACCGATT			
CAT	F: GCCCACACCGAGACCTTAA	183	102.64	Huang et al., 2016
	R: AACTTAGCTAAGCGGGGTACC			
GP_*x*_	F: ACTCCGCGCGTCCACG	149	97.04	Cheng et al., 2016
	R: GCCATTTCACCTTTGGAT			
CTSC	F: CAACGGGTTGGCCCGAGATCCCCAC	155	96.81	Designed by author
	R: ATTCTGGAAAACCCAACGGAAGT			
CTSD	F: CCTTACTTACGGGGTTCCCAAC	213	104.22	Designed by author
	R: GCTTTACCGGCGGCAAAAGGT			
HSP20	F: AACTTGCCAACCTCCTGACCTTTA	173	100.82	Designed by author
	R: GCCAAACCCTTACCAGGCAAAT			
HSP70	F: AACGGCTCTAAACGGAACTTGGG	189	97.05	Designed by author
	R: GGCCGTTTAACCCAGGGCTCT			
HSP90	F: GTAAAACCTCCAACAAAAGGCCCAGTT	134	99.31	Zheng et al., 2018
	R: CGAAAGCGCGGGCAAATCCGCAAGC			
β-Actin	F: ACACGGTAAAGCAACCTACC	207	102.92	Designed by author
	R: GCGCCCCAAACTTCCGAA			

*Mn-SOD, Mn-superoxide dismutase; CAT, catalase; GP_*x*_, glutathione peroxidase; CTSC, cathepsin C; CTSD, cathepsin D; HSP20, heat shock protein 20; HSP70, heat shock protein 70; HSP90, heat shock protein 90; F, forward primer; R, reverse primer.*

### Statistical Analysis

Logarithmic transformation was used to satisfy the homogeneity test of variances and standard normal distribution. One-way ANOVA (SPSS18.0) and Tukey’s test were utilized to analyze the difference of hemolymph osmotic pressure, ion concentration, activity of related enzymes, and gene expression in gill tissues under salinity changes at different sampling times, and *t*-test was performed to analyze the difference of associated physiological indexes in hemolymph and gill tissues between the salinity-changing group and the control group at the same sampling points, in which *P* < 0.05 was used as the significant difference level. All experimental data were indicated by mean ± standard error (mean ± SE) and the data after analysis were drawn using Sigmaplot.

## Results

### Survival Rate

**FIGURE 2:**
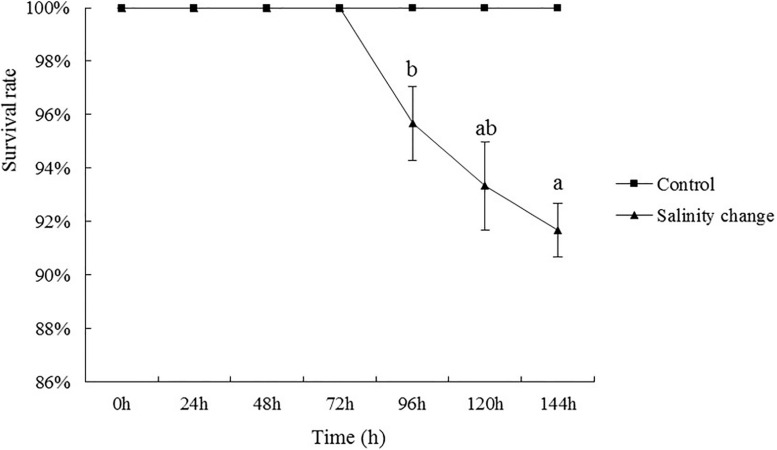
Effects of salinity changes on the survival rate of ark shell (*Scapharca subcrenata*). Values are expressed as mean ± SE (*n* = 4). Statistical analysis was performed by one-way analysis of variance (ANOVA) followed by Tukey’s test, using SPSS version 18.0. Means with the different lower case letters are significantly different at *P* < 0.05 level.

Throughout the experiment, no *S. subcrenata* were found dead in the control group. In the groups with sudden changes in salinity, the survival rate of *S. subcrenata* began to decrease at 96 h. The survival rate at the end of the experiment was 91.67%, although no significant difference was identified compared with 120 h (Figure 2, *P* = 0.281), which was significantly lower than that at any other time point during the study.

### Osmotic Pressure of Hemolymph Fluid

**FIGURE 3:**
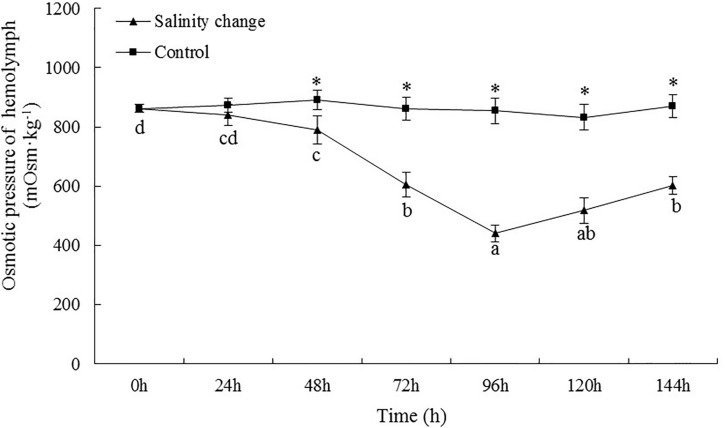
Effects of salinity changes on the osmotic pressure of hemolymph in ark shell (*Scapharca subcrenata*). Values are expressed as mean ± SE (*n* = 4). Statistical analysis was performed by one-way analysis of variance (ANOVA) followed by Tukey’s test, using SPSS version 18.0. Means with different lower case letters are significantly different at *P* < 0.05 level. Asterisks indicate significant differences between salinity change treatments and control treatment for the same time points, *P* < 0.05.

The osmotic pressure of the hemolymph in *S. subcrenata* changed in accordance with the changes in seawater salinity. At 96 h, the osmotic pressure of the hemolymph was not significantly different from that at 120 h (Figure 3, *P* = 0.175), but was significantly lower than that at any other time point during the study. At 144 h, when the salinity had increased to 30‰, the osmotic pressure concentration remained significantly lower than at 0, 24, and 48 h. Except at 0 and 24 h, the osmotic pressure concentration in the treatment group was significantly lower than in the control group at all other time points (48 h, *P* = 0.001; 72 h, *P* < 0.001; 96 h, *P* < 0.001; 120 h, *P* < 0.001; 144 h, *P* < 0.001).

### Concentrations of Na^+^, K^+^, Ca^2+^, and Cl^–^

**FIGURE 4:**
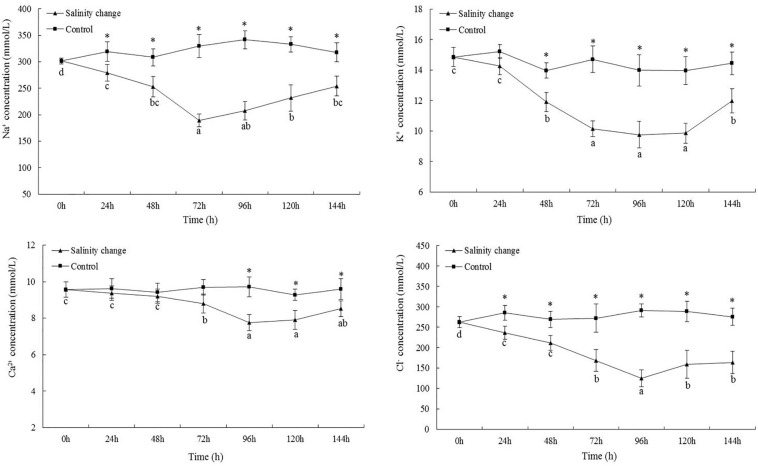
Effects of salinity changes on the Na^+^, K^+^, Ca^2+^, and Cl^–^ concentration of hemolymph in ark shell (*Scapharca subcrenata*). Values are expressed as mean ± SE (*n* = 4). Statistical analysis was performed by one-way analysis of variance (ANOVA) followed by Tukey’s test, using SPSS version 18.0. Means with the different lower case letters are significantly different at *P* < 0.05 level. Asterisks indicate significant differences between salinity change treatments and control treatment for the same time points, *P* < 0.05.

At 72 h, the Na^+^ concentration was not significantly different compared with 96 h (Figure 4, *P* = 0.142), but was significantly lower than that any other time point. Except at 0 h, the Na^+^ concentration in hemolymph from the treatment group was significantly lower than in the control group at all other time points (24 h, *P* = 0.003; 48 h, *P* < 0.001; 72 h, *P* < 0.001; 96 h, *P* < 0.001; 120 h, *P* < 0.001; 144 h, *P* < 0.001). The minimum K^+^ concentration occurred at 96 h, but was not significant different compared with 72 or 120 h (72 h, *P* = 0.183; 120 h, *P* = 0.269), although it was significantly lower than at any other point. The minimum concentration of Ca^2+^ also occurred at 96 h. At 144 h, the concentration of Ca^2+^ remained significantly lower than that at 0, 24, or 48 h (0 h, *P* < 0.001; 24 h, *P* = 0.001; 48 h, *P* = 0.001). At 96 h, the Cl^–^ concentration was significantly lower than that at any other time point. At 72, 120, and 144 h, there were no significant differences in the Cl^–^ concentration in the treatment group, but each was significantly lower than that at 0, 24, and 48 h. Except at 0 h, the concentration of Cl^–^ in the treatment group was significantly lower than in the control group at all other time points (24 h, *P* = 0.001; 48 h, *P* = 0.001; 72 h, *P* < 0.001; 96 h, *P* < 0.001; 120 h, *P* < 0.001; 144 h, *P* < 0.001).

### Hemoglobin, Soluble Total Protein, Free Amino Acids, and Moisture Content

Sudden changes in salinity had a significant effect on the concentration of hemoglobin in *S. subcrenata* (Table 2, *df1* = 6, *df2* = 21, *F* = 195.17, *P* < 0.001). At 96 h, the hemoglobin concentration peaked, and was significantly higher than at 0, 24, or 48 h (0 h, *P* < 0.001; 24 h, *P* < 0.001; 48 h, *P* < 0.001). The concentration of soluble total protein also peaked at 96 h, followed by a gradual decrease until 144 h, when it was significantly lower than at 96 h (*P* = 0.007). From 48 h to the end of the experiment, the content of soluble total protein and taurine in the treatment group was also significantly higher than in the control group. At 96 h, taurine content also peaked, and was significantly higher than at any other time point. As the salinity level decreased and then increased, the total free amino acid content tended to gradually increase and peaked at 144 h; although no significant difference was identified compared with 96 and 120 h (96 h, *P* = 0.258; 120 h, *P* = 0.327), each was significantly higher than at any other time point. The moisture content also tended to increase then decrease with the changes in salinity, with the moisture content of gill muscle peaking at 72 h, which was significantly higher than that at 0, 24, 48, and 144 h. From 72 to 120 h, the moisture content of muscle in the treatment group was significantly higher than in the control group (72 h, *P* < 0.001; 96 h, *P* = 0.001; 120 h, *P* = 0.001).

**TABLE 2:** Effects of sudden salinity changes on the contents of hemoglobin, soluble total protein, taurine, free amino acid, and tissue moisture in *Scapharca subcrenata*.

**Time**	**Treatment**	**Content**				
		**Hemoglobin (mmol/L)**	**Soluble total protein (mg/mL)**	**Taurine (mg/100 g)**	**Free amino acid (mg/100g)**	**Tissue moisture (%)**
0 h	Salinity change	1.420.21^b^	12.151.18^d^	56.528.72^f^	16.242.52^b^	73.091.42^b^
	Control	1.390.14	11.740.56	58.127.38	14.941.57	72.441.19
24 h	Salinity change	1.360.17^b^	14.591.26^c^	71.3313.59^e^	15.061.87^b^	72.261.75^b^
	Control	1.330.10	12.980.92	61.395.06	16.731.74	72.790.93
48 h	Salinity change	1.480.15^b^	15.861.15^c^	75.1810.64^e^	18.131.95^b^	74.511.82^b^
	Control	1.450.25	11.291.42*	57.3712.15*	16.112.64	71.020.75
72 h	Salinity change	2.370.23^a^	19.281.49^ab^	146.3715.26^c^	20.642.92^b^	82.091.94^a^
	Control	1.410.16*	13.511.18*	60.599.47*	18.791.38	71.890.88*
96 h	Salinity change	2.540.26^a^	22.352.07^a^	197.0216.90^a^	33.924.01^a^	81.551.43^a^
	Control	1.360.31*	12.090.63*	64.7510.15*	15.492.05*	73.140.52*
120 h	Salinity change	2.220.31^a^	20.141.73^ab^	166.4218.08^b^	35.163.87^a^	80.641.18^a^
	Control	1.390.20*	14.171.64*	61.647.97*	17.091.77*	72.961.03*
144 h	Salinity change	2.090.27^a^	18.662.13^b^	113.9214.14^d^	37.093.50^a^	75.921.52^b^
	Control	1.380.16*	12.851.02*	60.7910.74*	16.521.84*	71.740.72

*Values are expressed as mean ± SE (*n* = 4). Different lower case letters indicate significant differences in the contents of hemoglobin, soluble total protein, taurine, free amino acid, and tissue moisture for the different time points in salinity change treatments, *P* < 0.05. Asterisks indicate significant differences in the contents of hemoglobin, soluble total protein, taurine, free amino acid, and tissue moisture for the same time points between salinity change treatments and control treatment, *P* < 0.05.*

### Na^+^/K^+^-ATPase Activity, ROS Content, and T-AOC

**FIGURE 5:**
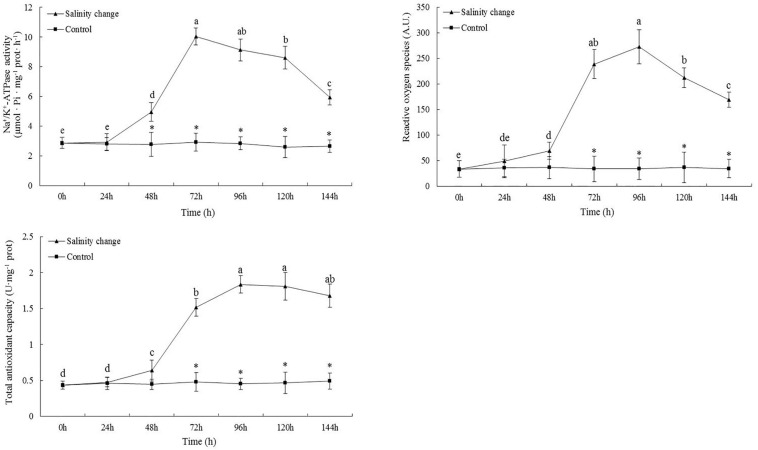
Effects of salinity changes on the activity of Na^+^/K^+^-ATPase, content of reactive oxygen species, and total antioxidant capacity in ark shell (*Scapharca subcrenata*). Values are expressed as mean ± SE (*n* = 4). Statistical analysis was performed by one-way analysis of variance (ANOVA) followed by Tukey’s test, using SPSS version 18.0. Means with different lower case letters are significantly different at *P* < 0.05 level. Asterisks indicate significant differences between salinity change treatments and control treatment for the same time points, *P* < 0.05.

At 48 h, the activity of Na^+^/K^+^-ATPase in the treatment group was significantly higher than in the control group and remained so until 144 h (Figure 5, **48** h, *P* = 0.002; 72 h, *P* < 0.001; 96 h, *P* < 0.001; 120 h, *P* < 0.001; 144 h, *P* < 0.001). Sudden changes in salinity also had a significant effect on the content of ROS (*df1* = 6, *df2* = 21, *F* = 163.26, *P* < 0.001). At 96 h, the content of ROS was at its maximum and remained significantly higher at 144 h compared with at 0, 24, and 48 h. At 96 h, T-AOC reached its maximum, which was significantly higher than at 0, 24, 48, and 72 h. From 72 to 144 h, T-AOC in the treatment group was significantly higher than in the control group (72 h, *P* < 0.001; 96 h, *P* < 0.001; 120 h, *P* < 0.001; 144 h, *P* < 0.001).

### Analysis of Gene Expression

**FIGURE 6:**
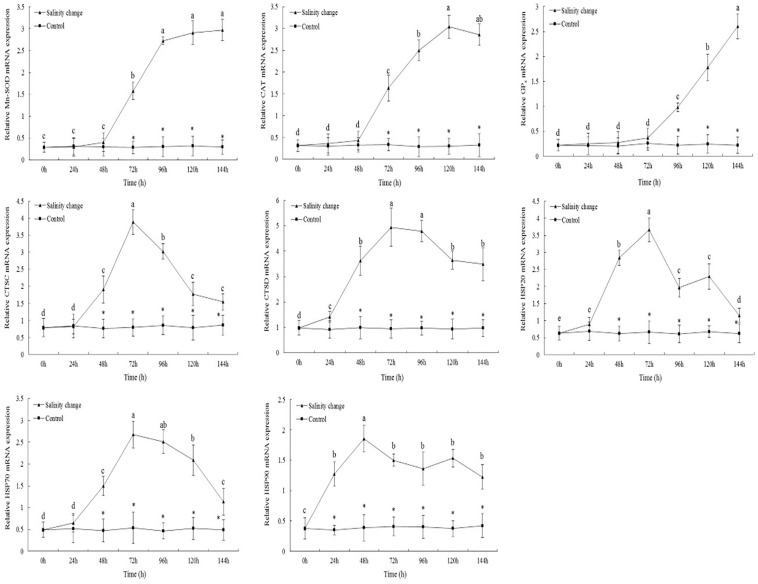
Relative mRNA expression levels of antioxidant enzyme and heat shock proteins’ genes in the gills of ark shell (*Scapharca subcrenata*). Values are expressed as mean ± SE (*n* = 4). Statistical analysis was performed by one-way analysis of variance (ANOVA) followed by Tukey’s test, using SPSS version 18.0. Means with different lower case letters are significantly different at *P* < 0.05 level. Asterisks indicate significant differences between salinity change treatments and control treatment for the same time points, *P* < 0.05.

Sudden changes in salinity had a significant effect on the expression levels of *MnSOD* (Figure 6, *df1* = 6, *df2* = 21, *F* = 227.18, *P* < 0.001). At 144 h, the expression level of *MnSOD* had peaked and was significantly different compared with 0, 24, 48, and 72 h. From 72 to 144 h, the expression levels of *MnSOD* in the treatment group were significantly higher than in the control group (72 h, *P* < 0.001; 96 h, *P* < 0.001; 120 h, *P* < 0.001; 144 h, *P* < 0.001). The expression levels of *CAT* tended to increase and then decrease with the sudden changes in salinity. At 120 h, no significant difference in the expression levels of *CAT* was identified compared with 144 h (*P* = 0.197), although it was significantly higher than at any other time point. At 144 h, the expression levels of *GP*_*x*_ were significantly higher than at any other time point. From 0 to 72 h, no significant difference in the expression levels of *GP*_*x*_ in the treatment group was identified compared with the control group; however, from 96 to 144 h, the expression levels of *GP*_*x*_ in the treatment group were significantly higher than in the control group (96 h, *P* < 0.001; 120 h, *P* < 0.001; 144 h, *P* < 0.001).

The maximum expression levels of *CTSC* occurred at 72 h, and were significantly lower at 96 h than at 72 h (Figure 6, *P* = 0.005), although both were significantly higher than at any other time point. From 48 to 144 h, the expression levels of *CTSC* and *CTSD* in the treatment group were significantly higher than in the control group. Sudden changes in salinity also had a significant effect on the expression levels of *CTSD* (*df1* = 6, *df2* = 21, *F* = 283.52, *P* < 0.001). At 72 and 96 h, the expression levels of *CTSD* were significantly higher than at any other time point.

With the sudden changes in salinity, the expression levels of *HSP20* tended to increase and then decrease, with the pattern then repeating. At 72 h, the expression levels of *HSP20* peaked and then decreased significantly after 96 h. From 48 to 144 h, the expression levels of *HSP20* in the treatment group were significantly higher than in the control group (Figure 6, **48** h, *P* < 0.001; 72 h, *P* < 0.001; 96 h, *P* < 0.001; 120 h, *P* < 0.001; 144 h, *P* = 0.014). The expression levels of *HSP70* also peaked at 72 h; although no significant difference was identified compared with 96 h, it was significantly higher than at any other time point. In the treatment group, the expression levels of *HSP90* were significantly higher than in the control group at 24 h (*P* < 0.001), and remained so until the end of the experiment. At 48 h, the expression levels of *HSP90* peaked, then decreasing, increasing, and then decreasing again, although the expression levels at each later time point were significantly lower than at 48 h.

## Discussion

The salinity level under which aquatic animals live varies greatly. For most aquatic animals, osmotic pressure regulation is a basic physiological process that enables the body to adapt to differences in internal and external ion concentrations. However, osmotic pressure regulation is complex because the environment inhabited varies from organism to organism (Sokolova et al., 2012; Kültz, 2015; Urbina and Glover, 2015).

The gill is the first organ to come into contact with the external environment. It has multiple physiological functions: in addition, to gas exchange, ammonia nitrogen excretion, acid–base balance regulation, it is an important site and primary organ for osmotic regulation (Politis et al., 2018; Giffard-Mena et al., 2020). Previous studies showed that the gill epithelial cells have a crucial role in ion regulation, in which Na^+^/K^+^-ATPase is vital to osmotic regulation by participating in the transport of ions (Abdel-Mohsen, 2009; Evans and Lambert, 2015). During the sudden changes in salinity in the current study, the Na^+^/K^+^-ATPase activity peaked at 72 h, when *S. subcrenata* was in 14‰ water for 24 h. As the salinity gradually returned to 30‰, the enzyme activity also gradually declined. Furriel et al. (2000) considered that Na^+^/K^+^-ATPase in the gills of crustaceans is the most important protease in osmotic regulation, Na^+^/K^+^-ATPase accounts for 70% of the total ATPase activity, and can transport Na^+^ out of the gill epithelial cells to the hemolymph, and transport K^+^ in the hemolymph into the gill epithelial cells, thereby maintaining the equilibrium of Na^+^ and K^+^ in body and the osmotic pressure of the hemolymph. When the salinity was 24‰, the expression levels of NKA mRNA in *Pagrus pagrus* larvae were significantly higher than in a group at a salinity of 34‰, and the osmotic pressure of the hemolymph showed the same trend with the changes in salinity (Ostrowski et al., 2011). When *Gadus morhua* was transferred from a salinity of 33 to 9‰, the expression of NKAα in the gills and kidney significantly reduced; in contrast, when transferred from 9 to 33‰, its expression levels significantly increased (Larsen et al., 2012). The activity of Na^+^/K^+^-ATPase in the gill of *Poecilia latipinna* following adaptation to a salinity of 35‰ was significantly higher than in the 15‰ or freshwater group; the content of NKAα subunit protein in the 35‰ salinity group was 2.2 and 1.7 times that in the 15‰ salinity and freshwater groups, respectively, and the osmotic pressure of the plasma showed the same trends (Yang et al., 2009). Therefore, Na^+^/K^+^-ATPase participates in the active transport of Na^+^ and K^+^ across the cell membrane and, thus, is important in regulating the osmotic pressure level of the hemolymph.

In response to external changes in salinity, aquatic organisms regulate the permeability of water and inorganic ions, and osmotic regulation involves changes in the content of osmotic effectors, such as inorganic ions and free amino acids, in the hemolymph. Inorganic ions have an important role in maintaining the osmotic pressure levels of the hemolymph, particularly Na^+^ and Cl^–^ (Cheng et al., 2002; McNamara and Faria, 2012). Crustaceans can actively absorb Na^+^ and Cl^–^ to compensate for salt loss from the body under low salinity levels, enabling them to adapt to environments with low or variable levels of salinity, such as estuaries (Flik and Haond, 2000). Inorganic ions are the main components affecting the osmotic pressure of the hemolymph in crustaceans. An increase in water salinity will increase the concentration of inorganic ions in the serum in *Eriocheir sinensis* and *Scylla serrate*, thus increasing its osmotic pressure (Romano et al., 2014; Long et al., 2017). In the treatment group, the concentrations of Ca^2+^ and K^+^ were significantly lower than in the control group at 96 and 48 h, respectively. These results also indicated that inorganic ions, such as Na^+^, Cl^–^, and K^+^, acted as primary osmotic effectors in the hemolymph and might have a greater impact on osmotic pressure compared with Ca^2+^. K^+^ plays a critical role in maintaining the osmotic pressure in neurons, maintaining the normal function of the nervous system. Intracellular changes of Na^+^ concentration affect the osmotic pressure response of organism, seal the mantle cavity, and protect cells from extreme salinity (Berger and Kharazova, 1997). Natochin et al. (1979) studied *Mytilus edulis* and *Littorina littorea* and found that, in addition to Na^+^ and K^+^ pumps, these organisms also have a Na^+^ exchange system related to Cl^–^ that regulates the cell volume and osmotic pressure. In this sense, the Na^+^–K^+^ pump and the Na^+^–Cl^–^ pump might jointly be involved enabling marine shellfish to cope with salinity changes.

Free amino acids in marine shellfish are also important osmotic effectors, and are mainly sourced from the decomposition of cells or hemolymph proteins. In crustaceans, with increases in environmental salinity, the serum protein decreases, whereas the content of free amino acids increases (Fu et al., 2017). Similar results have been found in shellfish placed in low salinity environments: when the cells swell because of the moisture produced in response to osmotic pressure differences, changes in plasma membrane permeability can induce the outflow of specific amino acids, thus enabling the cells to remove excess moisture and restore cell volume by the efflux of low molecular weight free amino acids (Silva and Wright, 1992, 1994). Taurine, glycine, and alanine are the most commonly used free amino acids, and the changes in taurine account for most of the free amino acid changes (Neufeld and Wright, 1996). In this study, the hemoglobin concentration peaked at 96 h, followed by a decrease, although it was still significantly higher than in the control group. In addition to carrying oxygen, hemoglobin can store energy and maintain osmotic pressure and antibacterial activity (Wang et al., 2013; Kato et al., 2017). Therefore, with sudden changes of salinity, because *S. subcrenata* lacks a specific immune system, it has to rely on the innate immunity of blood cells and humoral factors to enhance its resistance in response to external environmental stress.

For *C. gigas* under low osmotic conditions, most free amino acids in the mantle cells showed a significant, synchronous decrease within 2–8 h; under high osmotic condition, glycine, alanine, and taurine significantly increased, with the rapid increase in alanine having a crucial role in the short-term adaptation of salinity changes, whereas taurine was involved in long-term adaptation (Hosoi et al., 2003). In the current study, taurine and total free amino acids increased gradually with the sudden drop of salinity, with taurine peaking at 96 h when the salinity level had increased to 22‰, whereas total free amino acids increased gradually, suggesting that taurine has a crucial role in the rapid response of *S. subcrenata* to short-term sudden changes in salinity, whereas total free amino acids are involved in responses to long-term changes in salinity. Lu et al. (2015) reported that, when the salinity level of the external environment decreased, the content of total free amino acids in *Scylla paramamosain* also decreased, whereby the extent of the decrease in the content of glutamate, glycine, proline, and arginine accounted for 50% of the decrease in the total free amino acid content. The internal accumulation of certain free amino acids is a response to changes in external salinity. For osmotic pressure regulation, free amino acids can better stabilize macromolecules, such as proteins, compared with inorganic ions, without changing the structure and function of the enzyme or impacting the organism (Long et al., 2018). Therefore, free amino acids in the hemolymph are directly involved in the osmotic pressure regulation of *S. subcrenata*, and an effective means of maintaining the equilibrium of osmotic pressure is to increase the content of free amino acid.

Aerobic organisms continually produce ROS via metabolism. Excessive accumulation of ROS destroys macromolecular substances, such as proteins, carbohydrates, nucleic acids, and lipids (Finkel and Holbrook, 2000; Lushchak, 2011). As a result, maintaining the dynamic equilibrium between the production and removal of ROS is crucial to protect against oxidative damage and to maintain normal physiological functions (Martínez-Álvarez et al., 2005; Okoye et al., 2019). DCFH-DA shows sensitivity and specificity for the determination of ROS when used as a probe, but this type of compound is poorly selective against H_2_O_2_. Furthermore, the fluorescent response of DCFH and its derivatives is based on the oxidation mechanism, which responds to not only intracellular H_2_O_2_, but also other oxidation substances. It was also reported that an excessive concentration of DCFH can result in cytotoxicity, affecting the determination result (Miller et al., 2005; Wrona et al., 2005). In future studies, the method used to detect ROS should be selected by considering the low auto-oxidation, high optical stability, and low cytotoxicity of the method, in addition to its high selectivity and sensitivity.

However, aquatic animals have an evolutionarily conserved antioxidant defense system that can remove excess ROS. In particular, as antioxidant enzymes, SOD and CAT are considered to be the first line of defense against oxygen poisoning (Bhagat et al., 2016; Wang et al., 2020). SOD activities in the zebra and white zebra strains of *Ruditapes philippinarum* increased significantly with decreasing salinity from 30 to 5‰, with the highest value at a salinity of 5‰. CAT activity in the white and white zebra strains decreased with decreasing salinity from 30 to 5‰, but increased slightly in the zebra strain at 15‰ and then increased slightly at 5‰ (Nie et al., 2020). The current results showed that the expression levels of *MnSOD* and *CAT* in the treatment group at 72 h (equivalent to 24 h under a salinity level of 14‰) were significantly higher than in the control group, suggesting that sudden changes in salinity led to oxidative damage internally, with the continuously accumulated ROS requiring the organism to activate its antioxidant defense mechanisms before the dynamic equilibrium between oxidation and oxidation resistance could be restored. The expression levels of *MnSOD* at 144 h were still increasing, whereas the expression levels of *CAT* were lower than that at 120 h; thus, the higher expression levels of *MnSOD* might led to excessive accumulation of H_2_O_2_, thereby inhibiting the function of CAT. Nevertheless, as the second line of defense against oxidative damage, GP_*x*_ also has an important role in cell metabolism and scavenging of free radicals; in cells, it can catalyze the reduction of hydroperoxides to hydroxy compounds (Cnubben et al., 2001; Peña-Llopis et al., 2003). From 96 h onward, the expression levels of *GP*_*x*_ in the treatment group began to be significantly higher than in the control group. By the end of experiment, these were significantly higher than at any other point, whereas the expression levels of CAT tended to decrease; thus, it can be inferred that GP_*x*_ had a significant role in reducing toxic peroxides to non-toxic hydroxy compounds, thus shielding the structure and function of the cell membrane from interference and damage by the peroxide. However, when the expression levels of *CAT* decreased and that of *MnSOD* continued to increase, the scavenging of H_2_O_2_ was mainly dominated by *GP*_*x*_. Regarding enzyme kinetics, GP_*x*_ in mammals and other vertebrates has a greater affinity for H_2_O_2_ compared with CAT (Reddy et al., 1998; Avanzo et al., 2001). GP_*x*_ is thus largely responsible for the removal of H_2_O_2_ in vertebrates, while CAT and GP_*x*_ play complementary roles in H_2_O_2_ removal (Mourente et al., 2002; Jo et al., 2008). As a result, in response to sudden changes in salinity, CAT and GP_*x*_ had not only a synergistic, but also a dominant role in mediating the scavenging of H_2_O_2_ to maintain the dynamic equilibrium between internal oxidation and reduction.

In invertebrates, cathepsins act as a primary component of the lysosomal proteolytic system and are responsible for the specific degradation of intracellular proteins (Knop et al., 1993). As the salinity level decreased and then increased, the expression levels of *CTSC* and *CTSD* tended to increase and then decrease, peaking at 72 h (under a salinity of 14‰). Then, the expression levels of *CTSC* and *CTSD* decreased, although each was still significantly higher than in the control group. These changes suggested that, in response to salinity stress, the higher expression levels of *CTSC* and *CTSD* contributed to enhancing the innate immune response of the organism and its resistance to invasion and infection by pathogenic bacteria. Nair et al. (2005) studied *Strongylocentrotus purpuratus* and found that the expression levels of *CTSL* significantly increased subject to stimulation with lipopolysaccharide, thus inferring that *CTSL* is an important immune-related gene. Venier et al. (2006) found that, when *Mytilus galloprovincialis* was cultured in a stressful environment with heavy metals, the expression levels of *CTSL* were significantly higher than in the control group, suggesting that it is involved in the immune stress response. Therefore, our current findings indicated that cathepsins were involved in various physiological activities and had an important role in the regulation of innate immune response in invertebrates.

Heat shock proteins form a group of proteins with crucial physiological functions and a highly conserved structure; they are produced in large quantities in response to heat stress, tissue hypoxia, and osmotic pressure stress (Tine et al., 2010; Yang et al., 2019). In the current study, HSPs were induced with the sudden changes in salinity, although there were differences in the expression patterns of *HSP20*, *HSP70*, and *HSP90*. As a member of the small molecule HSP family, *HSP20* might not refold non-denatured proteins on its own, but might combine with unfolded proteins and other HSPs to form complexes that are involved in biological processes, such as cellular stress resistance, formation of germ cells, and development of tissues and organs (Morrow and Tanguay, 2012; Liu et al., 2013). Two peaks occurred in the expression levels of *HSP20*, at 72 h (under a salinity of 14‰) and at 120 h, suggesting that HSP20 had an important role in enabling the organism to adapt to changing salinity stress. Hsp70 has an important role in regulating the effects of environmental stress and maintaining homeostasis in cells (Casas and La Peyre, 2020; Hong et al., 2020). In the current study, the expression levels of *HSP70* peaked at 72 h, followed by a gradual decrease, although it remained significantly higher than in the control group at 144 h. This suggested that the continuously accumulated ROS led to the denaturation and degradation of partial proteins. At this point, the increased expression levels of *HSP70* contributed to the repair of damaged proteins and the effective regulation of the cell cycle. Salinity stress facilitated the increased expression levels of HSP70 in *A. japonicus*; especially under low salinity (20‰) stress, the higher expression levels of *HSP70* would help enhance the resistance of *A. japonicus* to salinity changes (Dong et al., 2008). Hsp90 is a molecular chaperone involved in protein maturation and has a role in maintaining the stability of protein construction, participating in cell cycle regulation, organism immunity, and signal transduction (Galt et al., 2018). In the current study, the expression levels of *HSP90* peaked at 48 and 120 h, and were significantly higher than in the control group from 24 h onward; thus, HSP90 might be more sensitive to sudden changes in salinity compared with HSP70 and HSP20, and had a specific role during the initial salinity changes, participating in signal transduction and maintained the stability of cell function. Wu et al. (2011) reported that the content of HSP90 mRNA stored in the cells for the maintenance of normal physiological function will be upregulated. It has also been shown that cysteine residues, methionine residues, and others in HSP90 are vulnerable to oxidation. HSP90 may be deprived of biological activity as molecular chaperone under long-term environmental stress. In the current study, the expression levels of HSP90 also began to fall after 72 h, then increased again until 120 h, but no significant difference was identified compared with 72 h. Thus, during the initial stages of responding to sudden changes in salinity, *S. subcrenata* enhanced signal transduction by relying on the high expression levels of *HSP90*. After 72 h, *S. subcrenata* relied more on the synergy of HSP20, HSP70, and HSP90 to better adapt to the environmental stress.

**FIGURE 7:**
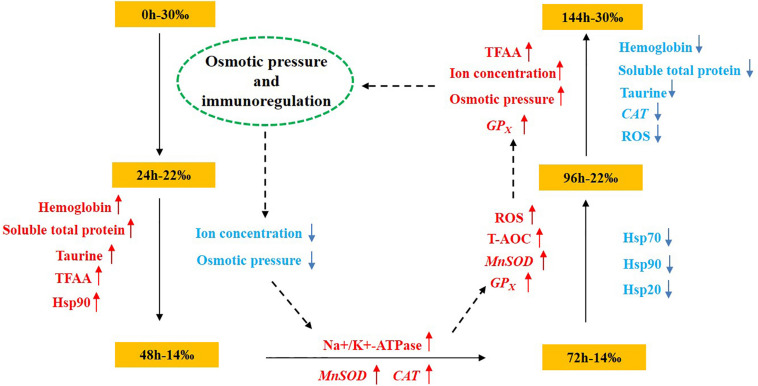
Schematic overview of the osmotic pressure and immunoregulation mechanism of *Scapharca subcrenata* in response to a sudden drop in salinity.

As an important economic shellfish in coastal and estuarine areas, seasonal heat and rainstorm often impact the survival of *S. subcrenata* after bottom sowing. In the current study, by simulating the sudden changes in salinity that occur after a rainstorm, it was found that the concentration of osmotic pressure of the hemolymph, and Na^+^, K^+^, Ca^2+^, and Cl^–^ content decreased and then increased with the changes in salinity, whereas hemoglobin, soluble total protein, taurine, and total free amino acid tended to increase with the decrease in salinity, with the content of hemoglobin, soluble total protein, and taurine then gradually decreasing as salinity increased (Figure 7). ROS peaked at 96 h, whereas the activity of Na^+^/K^+^-ATPase and the expression levels of *MnSOD*, *CAT*, *GP*_*x*_, *HSP20*, *HSP70*, and *HSP90* increased gradually. At 144 h, the expression levels of *MnSOD* and *GP*_*x*_ gradually increased, indicating that, although the salinity level had increased to 30‰, ROS had accumulated internally during the initial sudden changes in salinity. Thus, there was still a need for the antioxidant system to maintain the dynamic equilibrium between the production and removal of ROS, otherwise this would have impacted the cell structure and resulted in functional damage, which was an important cause of the reduced survival rate of *S. subcrenata* in the treatment group. Thus, there is a need to attempt to reasonably select the bottom-sowing and release area based on the osmotic pressure regulation mechanism of *S. subcrenata* and its tolerance to changing salinity levels and to avoid sowing and releasing after uninterrupted periods of rain, both approaches that will be instructive for improving the survival rate of *S. subcrenata* seedlings and, thus, the resource proliferation efficiency.

## Data Availability Statement

All datasets generated for this study are included in the article/supplementary material.

## Ethics Statement

All *S. subcrenata* in this study were handled in strict accordance with China legislation on scientific procedures on living animals. The protocol was approved by the ethics committee at University of Chinese Academy of Sciences (permit number: 399 20021109).

## Author Contributions

GX and LY conceptualized the study. ZM conducted research, collected the data, and wrote the manuscript. LL provided the materials and interpreted the data. GX had primary responsibility for the final content. All authors read and approved the final manuscript.

## Conflict of Interest

The authors declare that the research was conducted in the absence of any commercial or financial relationships that could be construed as a potential conflict of interest.
